# Zwitterionic chitosan for the systemic treatment of sepsis

**DOI:** 10.1038/srep29739

**Published:** 2016-07-14

**Authors:** Eun Jung Cho, Kyung-Oh Doh, Jinho Park, Hyesun Hyun, Erin M. Wilson, Paul W. Snyder, Michael D. Tsifansky, Yoon Yeo

**Affiliations:** 1 Department of Industrial and Physical Pharmacy, Purdue University, 575 Stadium Mall Drive, West Lafayette, IN 47907, USA; 2 Department of Physiology, College of Medicine, Yeungnam University, 317-1 Daemyung-dong, Daegu, Korea; 3 Department of Comparative Pathobiology, College of Veterinary Medicine, Purdue University, 625 Harrison Street, West Lafayette, IN 47907, USA; 4 Department of Pediatrics and the Congenital Heart Center, College of Medicine, University of Florida, 1600 SW Archer Road, Gainesville, FL 32610, USA; 5 Weldon School of Biomedical Engineering, Purdue University, West Lafayette, IN 47907, USA

## Abstract

Severe sepsis and septic shock are life-threatening conditions, with Gram-negative organisms responsible for most sepsis mortality. Systemic administration of compounds that block the action of lipopolysaccharide (LPS), a constituent of the Gram-negative outer cell membrane, is hampered by their hydrophobicity and cationic charge, the very properties responsible for their interactions with LPS. We hypothesize that a chitosan derivative zwitterionic chitosan (ZWC), previously shown to suppress the production of pro-inflammatory cellular mediators in LPS-challenged macrophages, will have protective effects in an animal model of sepsis induced by systemic injection of LPS. In this study, we evaluate whether ZWC attenuates the fatal effect of LPS in C57BL/6 mice and investigate the mechanism by which ZWC counteracts the LPS effect using a PMJ2-PC peritoneal macrophage cell line. Unlike its parent compound with low water solubility, intraperitoneally administered ZWC is readily absorbed with no local residue or adverse tissue reaction at the injection site. Whether administered at or prior to the LPS challenge, ZWC more than doubles the animals’ median survival time. ZWC appears to protect the LPS-challenged organisms by forming a complex with LPS and thus attenuating pro-inflammatory signaling pathways. These findings suggest that ZWC have utility as a systemic anti-LPS agent.

Severe sepsis (acute organ dysfunction secondary to infection) and septic shock (severe sepsis plus hypotension not reversed with fluid resuscitation) are life-threatening conditions frequently encountered in the intensive care units (ICUs) across the world^1^,^2^ and responsible for billions of dollars in medical costs every year in the United States^3^. As an infection overwhelms and disregulates the body’s defense mechanisms, sepsis may progress to shock, disseminated intravascular coagulation, and multiple organ failure^4^. Importantly, while some patients present to an ICU in severe sepsis or septic shock, others are admitted sepsis-free but develop an infection and progress along the above spectrum while being treated for something else. About 30–50% of septic shock patients die, even in the developed world^5^,^6^, and novel approaches to both therapy and prophylaxis of sepsis are urgently needed if we are to reduce this mortality.

The 2009 European Prevalence of Infection in Intensive Care (EPIC II) study has shown that Gram-negative organisms outnumber the Gram-positives as the etiology of septic shock, and that the only organisms associated with hospital mortality after multivariable logistic regression analysis are the *Enterococcus, Pseudomonas, and Acinetobacter* species^7^. The latter two species are, in fact, Gram-negative. Given this, efforts have been made to remove or inactivate the circulating lipopolysaccharide (LPS), a part of the outer cell membrane of Gram-negative bacteria and the primary trigger of the physiologic derangements seen in Gram-negative sepsis. LPS consists of a heteropolysaccharide chain and a glycolipid moiety (lipid A), which is partially phosphorylated (pKa 1.3) and negatively charged at neutral pH^8^. Lipid A is the most conserved and the most toxic part of LPS^9^,^10^; therefore, several compounds that can bind to lipid A via antigen-antibody, electrostatic, or hydrophobic interactions have been explored for the systemic treatment of sepsis^11^,^12^.

However, the very properties making these agents work well *in vitro* – their hydrophobicity and positive charge – limit their utility as a systemic therapy against Gram-negative sepsis. Polymyxin B, an amphiphilic polycationic peptide and one of the most effective neutralizers of LPS, is a potent nephro- and neurotoxin when given systemically^12^,^13^, mainly due to its cationic nature^14^,^15^. Other investigational anti-LPS agents such as LPS-binding proteins^16^,^17^,^18^, cationic peptide amphiphiles^19^,^20^, or cationic small molecules^10^,^21^,^22^,^23^ have also shown prohibitive systemic toxicity and/or non-specific protein binding^8^,^12^. Finally, systemic anti-LPS antibodies have failed in clinical trials partly due to non-specific hydrophobic interactions between them and irrelevant proteins^21^,^24^,^25^,^26^.

In this regard, it is worthwhile to note a chitosan (CS) derivative we reported previously^27^ as a potential alternative. This derivative, which we call zwitterionic chitosan (ZWC), is distinguished from other CSs, including the parent CS, in that it is negatively charged and water-soluble at the physiological pH due to the carboxyl groups introduced by partial succinylation. In our previous study ZWC showed excellent biocompatibility and no pro-inflammatory effect on naïve macrophages and uniquely suppressed the production of pro-inflammatory mediators in LPS-challenged macrophages^28^. Importantly, its anionic and hydrophilic nature protects ZWC from undesirable interactions with proteins or blood cells – a unique advantage over polycationic amphiphiles. Thus we hypothesized that ZWC might be suitable for systemic administration and investigated whether and how systemically-administered ZWC would mitigate the effects of LPS in mice.

## Results and Discussion

### Preparation of ZWC

**Figure 1:**
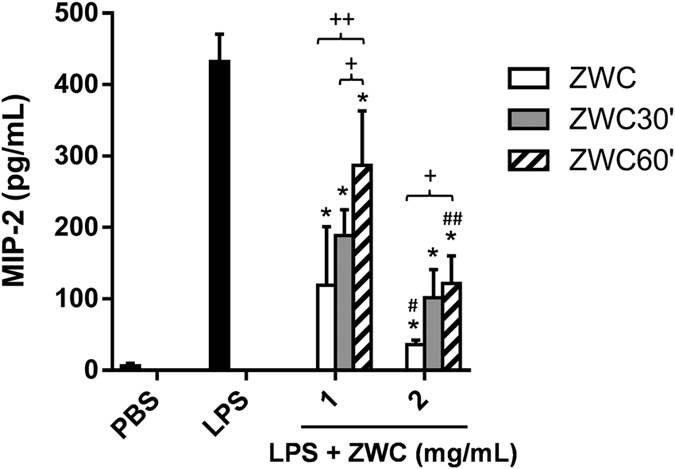
Dose dependent effects of ZWC, ZWC30’, and ZWC60’ on MIP-2 production from LPS-challenged PMJ2-PC mouse peritoneal macrophages. MIP-2 levels in the culture media of macrophages were determined by ELISA. The sampled media were diluted 10 times prior to analysis. Data are expressed as averages with standard deviations of three repeated measurements. *p < 0.0005 vs. LPS, ^#^p < 0.05 vs. 1 mg/mL; and ^##^p < 0.0001 vs. 1 mg/mL; ^+^p < 0.05; ^++^p < 0.0001 by Tukey test.

ZWC was produced by reacting CS and succinic anhydride with an anhydride to amine (An/Am) molar ratio of 0.7, because this product was superior to one made with a lower An/Am ratio in suppressing the production of a pro-inflammatory chemokine, macrophage inflammatory protein (MIP)-2, from LPS-challenged macrophages^28^. 53.4% of the repeating units were amidated at this ratio (Supplementary Fig. S1). ZWC had an isoelectric point (pI) of 4.5 (Supplementary Fig. S2) and showed good water solubility at pH’s distant from the pI, unlike the parent CS, which precipitated at pH 7 (Supplementary Fig. S3). Prior to the *in vivo* administration, ZWC was prepared in different molecular weights (MW) to find the optimal form. Lower MW ZWC’s were prepared by digesting ZWC (15 kDa) with H_2_O_2_ for different times (30 or 60 min). All ZWCs (ZWC, ZWC30’, and ZWC60’) inhibited MIP-2 production in LPS-challenged PMJ2-PC mouse peritoneal macrophages in a dose-dependent manner; however, the undigested ZWC was more effective than the degraded ones at each concentration (Fig. 1). ZWC was also produced with a higher MW CS (50–190 kDa) but had no effect on MIP-2 production, likely due to its limited solubility in the culture medium. Accordingly, we used 15 kDa ZWC for *in-vivo* administration.

### *In vivo* effects of ZWC in LPS-challenged mice

**Figure 2:**
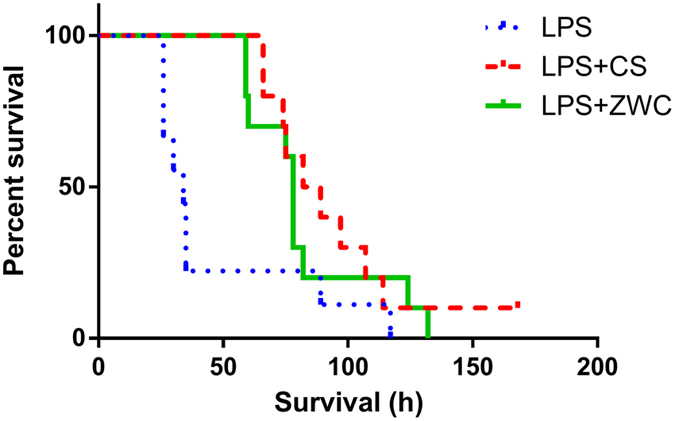
Kaplan–Meier analysis of survival. C57BL/6 mice were injected IP with LPS (20 mg/kg) and treatments (CS or ZWC, 800 mg/kg). n = 9 (LPS); n = 10 (CS, ZWC). LPS vs. LPS+ZWC: p = 0.0465, LPS vs. LPS+CS: p = 0.0634, by Log-rank (Mantel-Cox) test.

The protective effect of ZWC and CS was tested in a standard animal model of sepsis, where LPS is injected intraperitoneally (IP) to cause systemic inflammation that mimics the initial clinical features of sepsis, such as the production of pro-inflammatory cytokines, systemic hypotension, and decrease in glomerular perfusion^29^. C57BL/6 male mice were injected with LPS IP. ZWC or CS was administered IP together with LPS or 1 h prior to the LPS challenge, and the mice were observed for 1 week. Animals that received LPS indeed deteriorated quickly, showing acute hypothermia and weight loss (Supplementary Fig. S4). With no treatment, most animals died within 48 h, with a median survival time of 34 h (Fig. 2). On the other hand, animals receiving LPS simultaneously with ZWC or CS showed median survival times of 78 h and 85.5 h, respectively (LPS vs. LPS+ZWC: p = 0.0465, LPS vs. LPS+CS: p = 0.0634, by Log-rank (Mantel-Cox) test). Similarly, the treatment with ZWC or CS prior to the LPS challenge increased the median survival times to 74 h (ZWC) and 82 h (CS), although statistical difference from the LPS control was not observed due to the small sample size (Supplementary Fig. S5). In both simultaneous injection and pre-treatment, there was no significant difference between ZWC and CS-treated groups in the median survival time.

**Figure 3:**
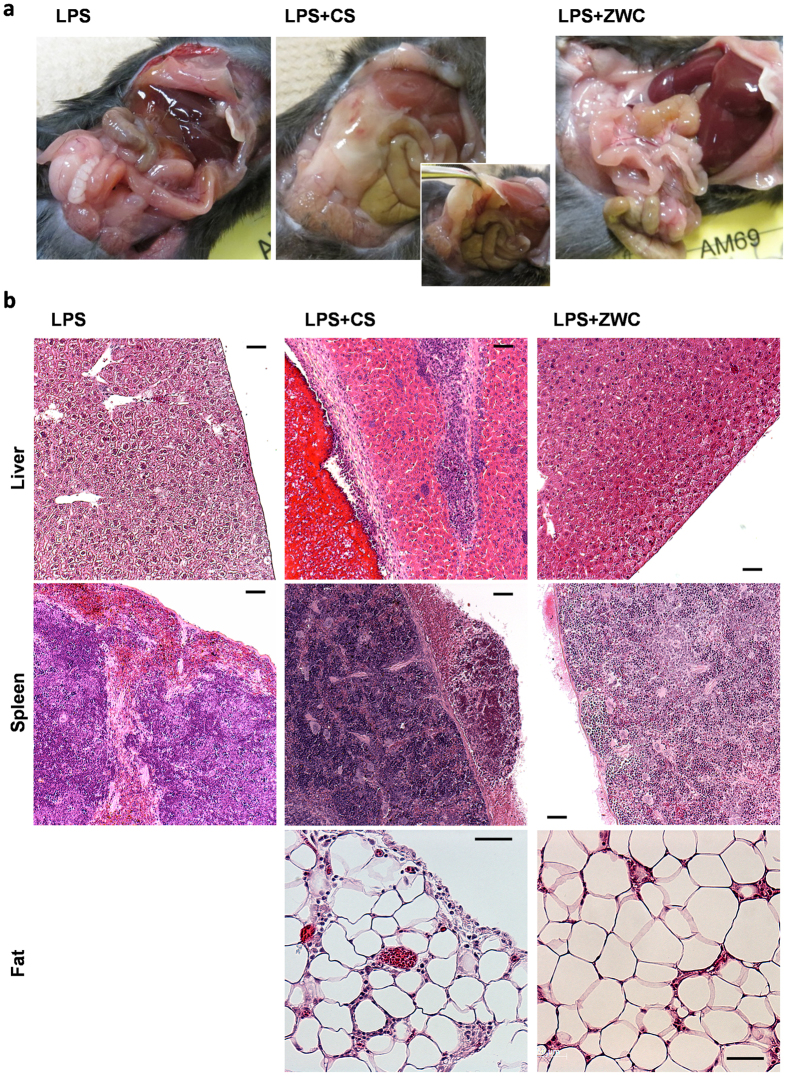
(**a**) Peritoneal cavities of mice injected with LPS only, LPS+CS, and LPS+ZWC. (**b**) Hematoxylin and eosin staining of liver, spleen, and fat sections of animals treated with LPS, LPS+CS and LPS+ZWC. Scale bar: 50 μm.

Although ZWC and CS appeared similarly effective in attenuating the effects of LPS *in vivo*, the tissue responses to these materials were different (Fig. 3a). Incidence of lesions in peritoneal tissues is summarized in Supporting Table 1. None of 9 LPS-challenged animals showed noticeable abnormalities or adhesions upon necropsy. Liver and spleen appeared grossly normal. In contrast, animals administered with CS (LPS+CS) showed adverse tissue responses to CS. Upon necropsy, 7 out of 10 animals had portions of the abdominal viscera (liver, spleen, intestine, kidney and mesentery) encased in a mass of fibrin with focal areas of hemorrhage. In all animals examined histologically, the serosal and capsular surfaces of the spleen, the liver, and the intestine were inflamed and contained copious amounts of fibrin, blood, neutrophils admixed with macrophages, and immature granulation tissue (Fig. 3b). Large collections of globular materials, presumably CS residues, were frequently associated with these changes. A similar but less severe reaction was present in the adjacent mesenteric fat. In the most severely affected regions, the inflammation extended into the muscular wall of the intestine or parenchyma of the liver (Fig. 3b). It is noteworthy that the organs connected to CS residues were those first exposed to the IP-injected solution, which indicates that CS precipitated out before it spread throughout the peritoneal cavity. On the other hand, there were no gross signs of adhesions or inflammation in any of the 9 ZWC-treated animals (LPS+ZWC). The absence of residual materials in the peritoneal cavities of the animals treated with ZWC suggests that the IP-injected ZWC was systemically absorbed via the peritoneal capillaries. Upon histological observation, only minimal multifocal collections of fibrin were present on the capsular surface of the spleen (Fig. 3b). The benign tissue responses and systemic absorption of ZWC, clearly unlike those for CS, are likely due to ZWC’s water solubility at neutral pH, which makes it desirable for systemic administration. This result is consistent with our previous observation of ZWC in healthy animals^28^.

### Mechanisms of ZWC action against LPS

**Figure 4:**
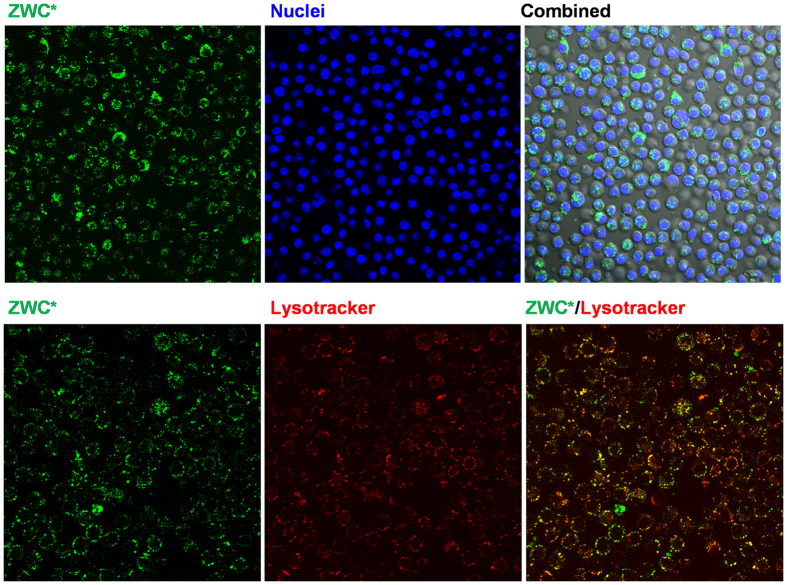
Confocal microscopy of PMJ2-PC mouse peritoneal macrophages treated with 0.6 mg/mL fluorescently labeled ZWC (ZWC*) for 3 h. Cell nuclei were stained with Hoechst prior to imaging. For lysosome staining, macrophages were first incubated with ZWC* and further incubated with 100 nM LysoTracker Red for 30 min.

While the *in-vivo* results in LPS-challenged animals show promise for ZWC as a systemic treatment of sepsis, its mechanism of action remains unclear. We previously attributed the anti-inflammatory effect of ZWC in the LPS-challenged macrophages to the extracellular interaction of LPS with ZWC^28^. On the other hand, we have also observed that ZWC entered macrophages and spread in the cytoplasm in 30 min, some co-localizing with lysosomes (Fig. 4). Therefore, we could not exclude an effect of ZWC on macrophages themselves. A series of experiments were performed to investigate extracellular and intracellular effects of ZWC on LPS.

#### Evidence for extracellular LPS-ZWC interaction

**Figure 5:**
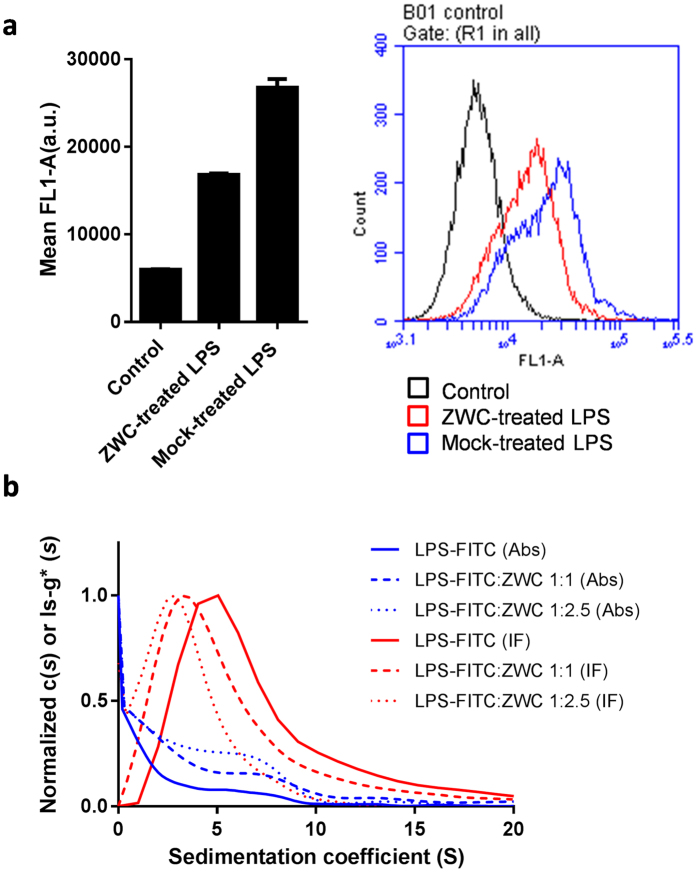
(**a**) Flow cytometry of PMJ2-PC mouse peritoneal macrophages treated with LPS-FITC. Gray: a control group with no treatment; red: a group receiving LPS-FITC pre-incubated with ZWC, precipitated at pH 4.8, centrifuged, and collected in the supernatant; blue: a group receiving mock-treated LPS-FITC. The graph on the left side shows averages and standard deviations of 3 measurements. All samples show significant difference from each other (Tukey test: p < 0.05). The plot on the right side shows a representative histogram. (**b**) Ls-g*(s) distribution of ZWC and LPS-FITC complexes. The interference (IF) signal distribution is attributable to both ZWC and LPS-FITC. Absorbance (Abs) signal at 495 nm confirmed the presence of LPS-FITC in the dominant species.

First, to confirm that ZWC directly interacts with LPS, fluorescently labeled LPS (LPS-FITC conjugate) was incubated with ZWC for 1 h. At the end of the incubation, ZWC was removed by precipitation at pH 4.8 (close to the pI value of ZWC), and the supernatant was incubated with PMJ2-PC mouse peritoneal macrophages. As shown in Fig. 5a, these macrophages displayed lower fluorescence intensity than those incubated with mock-treated LPS-FITC (treated in the same way without ZWC: reduction of pH, centrifugation, and collection of supernatant), indicating that there was less LPS-FITC in the supernatant. This suggests that LPS-FITC was removed together with ZWC, due to a direct interaction between ZWC and LPS-FITC.

Sedimentation coefficients (S) of LPS, ZWC, and LPS-ZWC mixtures, estimated by analytical ultracentrifugation (AUC), provided additional evidence for such an interaction. Here, LPS at a fixed concentration of 0.25 mg/mL was titrated with ZWC at increasing concentrations (0.25–1.25 mg/mL) and subjected to AUC. As shown in Table 1, the LPS-ZWC complex showed much lower sedimentation coefficients than that of LPS with the increase of ZWC concentration in the mixture, approaching the values of ZWC alone. In order to confirm that the peaks in the ls-g* distributions of LPS-ZWC mixtures contained LPS, LPS was replaced with LPS-FITC and the sedimenting boundary at 495 nm and the interference were monitored simultaneously. The ls-g* distribution indicated that LPS-FITC was present in the dominant species, with sedimentation coefficients of 7.8, 6, and 3.6 S according to the increase of ZWC concentration (Fig. 5b, Table 1). This suggests that LPS, which tends to form multimeric self-aggregates at a concentration above the critical value (13 μg/mL^30^), disaggregated in the presence of ZWC and underwent complexation/co-sedimentation with it. A similar observation was made with LPS-CS complexes by Yermak *et al.*^31^.

#### Nature of extracellular LPS-ZWC interaction

Although the results of flow cytometry and AUC suggest that ZWC directly interact with LPS, this interaction may not be explained by the main mechanisms by which conventional LPS antagonists (including CS) inactivate LPS. Electrostatic or hydrophobic interactions with LPS are not likely because ZWC is hydrophilic, anionic at neutral pH, and active in a cell culture medium with a physiological ionic strength. To prove this, surface plasmon resonance (SPR) was performed with ZWC and an L1 chip (GE Healthcare Life Sciences, Piscataway, NJ, USA), which had a surface composed of carboxymethylated dextran covalently conjugated with lipophilic groups (Supplementary Fig. S6a). Due to the negative charge of carboxymethylated dextran and hydrophobicity of lipophilic groups, the L1 chip served as an LPS-like platform to test the molecular binding of ZWC via electrostatic and hydrophobic interactions. According to the sensorgram, ZWC had little interaction with the L1 chip irrespective of the concentration, while a positive control (1,2-dimyristoyl-sn-glycero-3-phosphoethanolamine-N-[methoxy(polyethylene glycol)-550], PEG_550_-PE) flowed at the same rate and time showed significant binding to the chip (Supplementary Fig. S6b). Having excluded electrostatic or hydrophobic interactions, the most likely mechanism of the ZWC-LPS interaction is the hydrogen bond between -NH_2_ and -NH-(C=O)-CH_2_-CH_2_-(C=O)-OH) groups of ZWC and the lipid A phosphates^21^. It is possible that ZWC is a more robust former of the hydrogen bond than CS, since each succinylation brings two more H-bond acceptor/donors (NH-*H* vs. NH-(C=*O*)-CH_2_-CH_2_-(C=*O*)-*OH*).

#### ZWC effects on macrophage activation

Macrophages are the main effectors of innate immunity, responsible for the initial pro-inflammatory phase of sepsis upon systemic exposure to LPS^32^,^33^. Given the evidence of ZWC entry into macrophages (Fig. 4), we suspected that ZWC may have direct effects on LPS-induced intracellular signaling in macrophages. This involves LPS binding to a receptor complex composed of CD14, toll-like receptor 4 (TLR4), and MD2, which triggers signal propagation via the IκB kinase (IKK) and mitogen activated protein kinase (MAPK) pathways, leading to activation and nuclear localization of NF-κB and AP-1 and production of pro-inflammatory cytokines^34^,^35^. Previous studies have shown that pre-treatment with CS oligosaccharides interferes with MAPK signaling in endothelial cells^36^ and RAW264.7 macrophages^37^, thereby inhibiting LPS-induced IL-6 production in those cells. A similar result was obtained with the RAW264.7 macrophages pre-treated with another water-soluble derivative of CS and then challenged with an allergen^38^. Opinions on how the CS derivatives suppress the LPS-initiated signaling events vary. While Wang *et al.* proposed that the CS effect was restricted to intracellular signaling^38^, Du *et al.* demonstrated that CS oligosaccharides inhibited LPS binding to a TLR4/MD-2 receptor complex of RAW264.7 macrophages, thereby attenuating subsequent signaling pathways^39^. As a derivative of CS, ZWC may have a similar effect on LPS-macrophage binding and/or intracellular signaling; therefore, we tested its effect on both.

**Figure 6:**
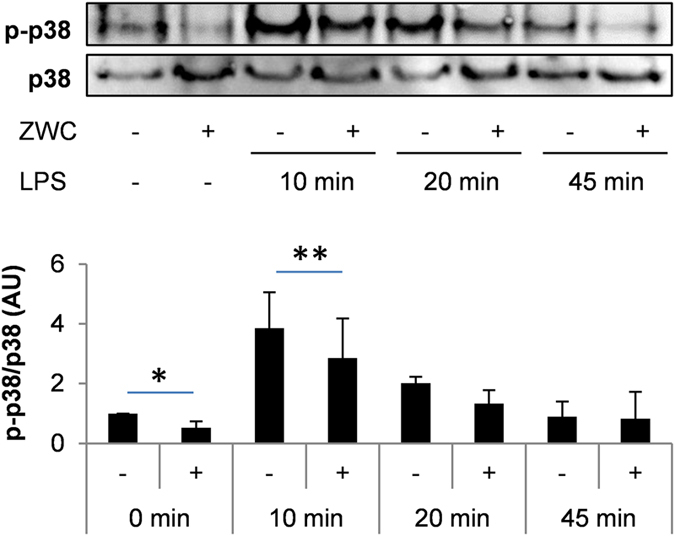
Inhibitory effects of ZWC on LPS-induced phosphorylation of p38 in macrophages. PMJ2-PC mouse peritoneal macrophages were incubated with or without 2 mg/mL of ZWC overnight and challenged with 1 μg/mL of LPS for 10, 20, or 45 min after removing excess ZWC. Western blotting was performed with macrophage cell lysates. The bar graph indicates the band intensity normalized by the intensity of total p38 using ImageJ. Data are expressed as averages and standard deviations of 3 independently and identically performed experiments. *p < 0.05, **p < 0.01 by one-tailed paired t-test.

To investigate the ability of ZWC to interfere with LPS binding to macrophages and subsequent internalization, PMJ2-PC mouse peritoneal macrophages were incubated with LPS-FITC simultaneously with ZWC or after pre-treatment with ZWC. Simultaneous incubation would mainly probe whether LPS-binding to macrophages is inhibited due to the LPS-ZWC complexation shown in Fig. 5, whereas pre-treatment would determine whether ZWC competes with LPS for the same receptor. Flow cytometry found little difference in the FITC level in macrophages treated with LPS-FITC, whether they were treated with LPS-FITC alone, LPS-FITC and ZWC simultaneously, or pre-treated with ZWC prior to LPS-FITC addition (Supplementary Fig. S7). This indicates that neither the formation of LPS-ZWC complex nor ZWC itself interferes with the LPS binding to macrophages. To investigate the effect of intracellular ZWC on LPS-induced signaling, we examined the phosphorylation of p38, a prominent member of the MAPK family, in macrophages treated with LPS and/or ZWC. To focus on the intracellular effect of ZWC, we performed an LPS challenge on macrophages pre-treated with ZWC (i.e., macrophages that had internalized ZWC) in the absence of excess extracellular ZWC. As shown in Fig. 6, the phospho-p38 (p-p38) level in LPS-challenged macrophages increased at 10 min and returned to the basal level at 45 min, consistent with the literature^36^; meanwhile, the ZWC-pre-treated macrophages showed a significantly reduced levels of p-p38 at 10 min, suggesting inhibitory effect of ZWC on LPS-initiated signaling pathways.

In summary, this study shows that ZWC, a partially succinylated CS derivative, provided a protective effect in a mouse model of LPS-induced shock when given simultaneously with or prior to the LPS challenge. Due to its water solubility at physiological pH, the IP-injected ZWC was readily absorbed with no local residues or adverse tissue reactions at the injection site, unlike the parent CS. ZWC appeared to protect macrophages from the LPS challenge by forming a complex with LPS, thus attenuating pro-inflammatory signaling pathways. Taken together, our findings suggest that ZWC may have utility as a systemic anti-LPS agent. While the effect of ZWC administered after the onset of sepsis (the more clinically relevant scenario for the treatment paradigm) remains to be seen, its potential for sepsis prophylaxis (*e.g.,* given to all high risk inpatients before the onset of sepsis) is quite promising.

## Materials and Methods

### Materials

Chitosan (CS; MW: 15 kDa; degree of deacetylation: 87%) was purchased from Polysciences (Warrington, PA, USA). LPS, LPS-FITC conjugate, and CS with a molecular weight of 50–190 kD and a deacetylation degree of 83% were purchased from Sigma-Aldrich (St. Louis, MO, USA). FPR-648 dye was a gift from BioActs (Incheon, Korea). PMJ2-PC mouse peritoneal macrophage cell line was purchased from ATCC (Manassas, VA, USA). Macrophage inflammatory protein (MIP)-2 enzyme-linked immunosorbent assay (ELISA) kit was purchased from R&D Systems (Minneapolis, MN, USA). LysoTracker Red DND-99, cell culture medium and supplements were purchased from Invitrogen (Carlsbad, CA, USA). p38 MAPK and p-p38 MAPK primary antibodies and HRP-conjugated anti-rabbit IgG were purchased from Cell Signaling Technology (Danvers, MA, USA). All other reagents were purchased from Sigma-Aldrich.

### ZWC synthesis

ZWC was produced as reported previously^27^,^28^. Briefly, 200 mg of CS acetate was dissolved in 30 mL of water, and 70 mg of succinic anhydride (anhydride to amine ratio, An/Am ratio of 0.7) was added as solid to the CS solution while stirring. The reaction mixture was maintained at pH 6–6.5 for 1 h, stirred overnight at pH 8–9, and dialyzed against deionized water prior to lyophilization. Optionally, ZWC was reacted with 30% H_2_O_2_ under vigorous stirring for 30 or 60 min at room temperature to produce lower molecular weight ZWC (named ZWC30’ and ZWC60’ according to the reaction time)^40^. For quality control of ZWC, the zeta potential of ZWC solution was measured at different pH’s, the pI determined, and H-NMR spectra examined as described in our previous report^27^.

### MIP-2 production assay from LPS-challenged macrophages

PMJ2-PC mouse peritoneal macrophages were grown in Dulbecco’s Modified Eagle Medium (DMEM) supplemented with 5% fetal bovine serum, 5 mM HEPES, 100 units/mL of penicillin and 100 μg/mL of streptomycin (referred to as complete medium). The cells were seeded in a 24-well plate at a density of 150,000 cells per well in 1 mL of medium. After overnight incubation, LPS (from *Escherichia coli* O111:B4) was added to the medium in the final concentration of 1 μg/mL. Subsequently, 100 μL of ZWC solution was added to each well to bring the final chitosan concentration in the medium to 1 or 2 mg/mL. In control groups, PBS was added in lieu of ZWC solution. After a 24-h incubation, the plate was centrifuged at 931 rcf for 10 min to separate culture medium from the cells. The concentration of macrophage inflammatory protein (MIP)-2 in the medium was determined using an MIP-2 ELISA kit according to the manufacturer’s instruction. A standard calibration curve was prepared in the range of 0–500 pg/mL. The sampled medium was diluted 10 times prior to the ELISA analysis.

### Confocal microscopy of macrophage uptake of ZWC

ZWC was fluorescently labeled for tracking its uptake by macrophages. Twenty five milligrams of ZWC was dissolved in 2.5 mL of 0.1 M NaHCO_3_ buffer (pH 9.0) and mixed with 100 μL of 10 mg/mL aqueous FPR-648 dye solution (λ_Ex_: 648 nm; λ_Em_: 672 nm). The mixture was reacted overnight in darkness. The fluorescently labeled ZWC (ZWC*) was purified by dialysis against deionized water and lyophilized. Peritoneal macrophages were plated in 35 mm dishes at a density of 160,000 cells/cm^2^. After 24 h, the medium was replaced with 1 mL of fresh complete medium containing 0.6 mg/mL ZWC*. After 3 h of incubation with, cells were washed twice with the medium in order to remove the free ZWC*. When lysosomes were stained, the ZWC*-laden cells were incubated in 100 nM LysoTracker Red for 30 min. After washing, Hoechst 33342 was added to 2 μg/mL 30 min prior to imaging. Confocal microscopy was performed using Nikon A1R confocal microscope equipped with a Spectra Physics 163C argon ion laser and a Coherent CUBE diode laser. ZWC* was excited with a 640 nm laser, and the emission was read from 660 to 710 nm. Cell nuclei were excited with a 408 nm laser, and the emission was read from 425 to 475 nm. LysoTracker was excited with a 561 nm laser, and the emission was read from 570 to 620 nm.

### Western blotting

PMJ2-PC peritoneal macrophages were seeded in 24-well plate with a seeding density of 1.5 × 10^5^ cells per well in 1 mL of complete DMEM. After an overnight incubation, one tenth of medium was replaced with PBS (control group) or PBS containing 2 mg of ZWC (treatment group). After 20 h of incubation, the cells were centrifuged at 335 rcf for 5 min. After discarding the supernatant, the cells were redispersed in fresh complete medium containing 1 μg/mL LPS and incubated for 10, 20, or 45 min. The cells were then harvested and lysed in 0.25 mL of protein solubilizing mixture containing 25% sucrose, 2.5% sodium dodecyl sulfate (SDS), 25 mM Tris, 2.5 mM EDTA and 2.5% pyronin Y. Forty microliters of cell lysate was separated in 10% SDS-polyacrylamide gel and transferred to polyvinylidene fluoride membranes. The membranes were blocked with 0.5 v/v% goat serum in NP40 buffer for 0.5 h and incubated with p38 MAPK and p-p38 MAPK primary antibodies overnight at 4 °C. The antibodies were detected with HRP-conjugated anti-rabbit IgG for 1 h at room temperature. Immunoreactive bands were visualized with enhanced chemiluminescence reagents (ECL) and detected by Azure C300 (Azure Biosystems, Inc., Dublin, CA, USA).

### Flow cytometry

Flow cytometry was performed on peritoneal macrophages incubated with fluorescently labeled LPS (LPS-FITC) for different purposes. To test whether ZWC interacts with LPS, 50 μg of LPS-FITC was mixed with 10 mg of ZWC in 1 mL of 0.9% NaCl and incubated at room temperature for 1 h. ZWC was then precipitated by decreasing the solution pH to 4.8 with 0.1 M HCl and removed by a 15-min centrifugation at 9,300 rcf. Assuming that LPS-FITC was present in the supernatant, a volume of supernatant equivalent to 1 μg of LPS-FITC was sampled and added to 1 mL of the peritoneal macrophage culture in the complete medium. LPS-FITC treated in the same way without ZWC (mock-treated) was used for a control group of cells. After 10 h of incubation, cells were collected by gentle pipetting and analyzed with a BD Accuri C6 flow cytometer (San Jose, CA, USA).

To test whether the ZWC-LPS interaction interferes with LPS binding to macrophages, LPS-FITC was added to macrophages together with ZWC, bringing their concentrations in culture to 2 μg/mL and 2 mg/mL, respectively, and incubated for 1 or 2 h at 37 °C. To test whether ZWC competes with LPS for the same receptor, the macrophages were pre-treated with 2 mg/mL of ZWC for 1 h prior to the addition of LPS-FITC. After a 2-h incubation, the macrophages were collected and analyzed with a Beckman Coulter FC500 flow cytometer (Indianapolis, IN, USA). LPS-FITC-bound macrophages were detected with an FL1 detector (λ_Ex_: 488 nm; λ_Em_: 525/40 nm). For all analyses, untreated cells were used as a negative control. A total of 10,000–20,000 gated events were acquired for each analysis.

### Analytical ultracentrifugation

To elucidate the shape distributions of ZWC and LPS and their interactions, sedimentation velocity experiments were conducted on a Beckman Coulter XLI analytical ultracentrifuge. LPS (or LPS-FITC) and ZWC samples were mixed and dialyzed extensively against PBS buffer at room temperature. LPS concentration was kept constant at 0.25 mg/mL, whereas ZWC concentration was varied from 0.25 to 1.25 mg/mL. The samples were then centrifuged at 201,600 or 32,256 rcf using two-sector 1.2 cm path-length carbon-filled Epon centerpieces. The experiments were conducted on an An-50 Ti rotor at 20 °C. Interference scans were collected every five minutes for a total of 150 scans. LPS-FITC was measured at 495 nm in absorbance in addition to interference optics. The density and relative viscosity of the buffers were calculated with SEDNTERP version 20120828 BETA^41^ to be 0.99823 g/mL and 0.01018 P, respectively. ls-g* distributions were analyzed using SEDFIT version 14.3e^42^.

### Surface plasmon resonance

SPR analysis was performed using a Biacore 3000 (GE Healthcare Life Sciences, Piscataway, NJ, USA) to detect the ability of ZWC to establish electrostatic and/or hydrophobic interactions with a surface. An L1 sensor chip with negatively charged carboxymethylated dextran and hydrophobic alkyl chains was used as a model surface. ZWC was dissolved in HEPES-buffered saline (HBS, pH 7.4) at a concentration of 10 μM or100 μM and injected for 5 min at a flow rate of 4 μL/min. As a positive control, PEG550-PE was injected at a concentration of 0.5 mM for 5 min. The L1 chip was regenerated using 40 mM n-octyl β-D-glucopyranoside prior to each injection. The running buffer was HBS, and experiments were performed at 25 °C.

### Administration of ZWC in septic animals

All animal procedures were performed according to a protocol approved by the Purdue Animal Care and Usage Committee, in accordance with the NIH Guideline for the Care and Use of Laboratory Animals. Male C57BL/6 mice at 8–9 weeks of age weighing 24.8 ± 1.5 g were used for this study. The animals were kept at 25 °C with 12 h light-dark cycles, and food and water were allowed *ad libitum*. After a one-week acclimatization period, the mice were randomly divided into LPS (n = 9), ZWC (n = 10), and CS groups (n = 10). The animals in the LPS group received an IP injection of the LPS (*E. coli* O111:B4, 20 mg/kg) solution in 1 mL of sterile saline, and those in the ZWC and CS groups received a mixture of LPS (20 mg/kg) and ZWC or LPS and CS (800 mg/kg) in 1 mL of sterile saline. For the observation of pre-treatment effect, ZWC or CS (800 mg/kg) was injected IP 1 h prior to the LPS injection (n = 5 for each group). The animals were observed every 6–8 h up to 1 week. The body temperature was measured with a Pocket Infrared Thermometer (Braintree Scientific, Inc., Braintree, MA, USA) at each observation, and the body weight recorded daily. Buprenorphine (0.05 mg/kg) was injected subcutaneously every 6–8 h for 2 days and when severe signs of distress (labored breathing, hunched positioning, and reluctance to move) were observed. When an animal was found dead at the time of observation, the time of death was estimated to be in the middle of the last two observation times. When an animal was found to be moribund at the time of observation, animals were euthanized by CO_2_ asphyxiation followed by cervical dislocation. Upon necropsy, organs in the peritoneal cavity were sampled, fixed in 4% formalin, and embedded in paraffin for hematoxylin and eosin staining.

### Statistical analysis

All data were expressed as means ± standard deviations. Statistical analyses were performed with GraphPad Prism 6 (La Jolla, CA, USA). Unless specified otherwise, one-way ANOVA was performed to determine the difference among the groups, followed by pairwise comparison based on the Tukey procedure. *In-vivo* survival data were plotted using the Kaplan-Meier method and analyzed with the Log-rank (Mantel-Cox) test. A value of p < 0.05 was considered statistically significant.

## Additional Information

**How to cite this article**: Cho, E. J. *et al.* Zwitterionic chitosan for the systemic treatment of sepsis. *Sci. Rep.* **6**, 29739; doi: 10.1038/srep29739 (2016).

## Supplementary Material

Supplementary Information

## Figures and Tables

**Table 1:** Sedimentation coefficients of LPS:ZWC mixtures.

Sample	LPS (mg/mL)	ZWC (mg/mL)	Sedimentation coefficient (S)
LPS	0.25	0	3.3, 15, 46
ZWC	0	0.25	2.1
ZWC	0	0.625	2
ZWC	0	1.25	2
LPS:ZWC (1:1)	0.25	0.25	2.1
LPS:ZWC (1:2.5)	0.25	0.625	2
LPS:ZWC (1:5)	0.25	1.25	2
LPS-FITC	0.25	0	7.8
LPS-FITC:ZWC (1:1)	0.25	0.25	6
LPS-FITC:ZWC (1:2.5)	0.25	0.625	3.6

LPS, ZWC, and LPS:ZWC mixtures were spun at 201,600 rcf, and LPS-FITC and LPS-FITC:ZWC mixtures were at 32,256 rcf.
