# Supplementary material for: Zwitterionic chitosan for the systemic treatment of sepsis
Source: Sci Rep. 2016 Jul 14;6:29739. doi: 10.1038/srep29739 (PMC4944199; doi:10.1038/srep29739)
Supplement: Supplementary Information [file srep29739-s1.doc]

Supplementary Information

Zwitterionic chitosan for the systemic treatment of sepsis

Eun Jung Cho^†^, Kyung-Oh Doh^†^, Jinho Park^†^, Hyesun Hyun, Erin M. Wilson, Paul W. Snyder, Michael D. Tsifansky, and Yoon Yeo^*^

**Supplementary Fig. S1**. ^1^H NMR spectra and peak assignments of unmodified chitosan (CS) and ZWC produced with an An/Am ratio of 0.7 (ZWC_0.7_).

**Supplementary Fig. S2.** pH-dependent zeta potential profile of ZWC. The curve is drawn with average values of three measurements.

**Supplementary Fig. S3.** Difference in water solubility between CS and ZWC_0.7_. At acidic pH, both CS and ZWC0.7 dissolve in water. At pH 7, CS does not dissolve but ZWC_0.7_ dissolves in water.

**Supplementary Fig. S4.** Body weights and temperatures (measured by an infrared thermometer) of surviving C57BL/6 male mice IP injected with LPS (20 mg/kg) and treatments (CS or ZWC, 800 mg/kg). Note that there is ~7 °C difference between a rectal thermometer and an infrared thermometer due to the attenuation by hair and air. An average body temperature of a normal mouse measured with the infrared thermometer is 30.6 ± 0.5 °C. The reported rectal temperature of C57BL/6 mice (2 months) is 37.4 ± 0.3 °C.[^1^](#_ENREF_1)

1. Habicht, G. S. Body temperature in normal and endotoxin-treated mice of different ages. *Mech. Ageing Dev.* **16**, 97-104 (1981).

**Supplementary Fig. S5.** (a) Kaplan–Meier analysis for survival. C57BL/6 male mice were injected IP with LPS (20 mg/kg) as well as pre-treatment with CS or ZWC (800 mg/kg). n=9 (LPS); n=4 (pre-treated with CS or ZWC). No statistical difference between LPS vs. pretreated with CS or ZWC by Log-rank (Mantel-Cox) test. (b) Body weights and (c) temperatures of surviving C57BL/6 mice injected with LPS (20 mg/kg) and pre-treatment with CS or ZWC (800 mg/kg). Note that there is ~7 °C difference in body temperatures measured with a rectal thermometer and an infrared thermometer due to the attenuation by hair and air. An average body temperature of a normal mouse measured with the infrared thermometer is 30.6 ± 0.5 °C. The reported rectal temperature of C57BL/6 mice (2 months) is 37.4 ± 0.3 °C.[^1^](#_ENREF_1)

**Supplementary Fig. S6.** (a) Diagram of L1 chip. (b) Sensogram depicting the interaction of ZWC with L1 chip. 10, 100 µM of ZWC was injected, which resulted in an increase of < 100 relative response unit. PEG550-PE was injected, which resulted in an increase of 1000 response unit. The surface was regenerated by a pulse of 40 mM n-octyl β-D-glucopyranoside. At all times, the flow rate was maintained at 4 µL/min.

**Supplementary Fig. S7**. Flow cytometry of peritoneal macrophages treated with LPS-FITC. Gray: a control group with no treatment; red: a group receiving LPS-FITC only (2 µg/mL); green: a group pre-treated with 2 mg/mL of ZWC for 1 h prior to LPS-FITC incubation; blue: a group treated with a mixture of ZWC and LPS-FITC prepared 1 h ahead.

**Supplementary Table 1**. Incidence of lesions in tissues after intraperitoneal injection of LPS, LPS+CS, or LPS+ZWC.

|  | LPS | LPS+CS | LPS+ZWC |
| --- | --- | --- | --- |
| Macroscopic abnormalities (adhesion or material residues) | 0/9 | 7/10 | 0/10 |
| Liver, capsular inflammation | 0/1 | 3/4 | 0/3 |
| Spleen, capsular inflammation | 0/1 | 2/2 | 0/1 |
